# Advertising

**Published:** 1898-07

**Authors:** H. C. Sexton

**Affiliations:** Shelbyville, Ind.


ARTICLE VIII.

### ADVERTISING.

H. C. SEXTON, D. D. S., SHELBYVILLE, IND.

I cannot help but feel that a person better suited to write on this subject could have been secured by your committee, but having undertaken the writing of a paper on advertising, I will give you my ideas regarding it.

These ideas have been developed and confirmed in my mind during the nine years I have practiced. Experience is everywhere recognized as a thorough teacher, and my ideas, I do not hesitate to say, were to a great extent gained by experience. Therefore, in considering my paper, I hope that your attention will rest on the thoughs introduced, and not upon the literary dressing of the production, for as an essay, considered purely from a literary standpoint, you will find it very imperfect.

I will speak first of that mass of advertising which we daily see and which is so offensive to good taste. This class of advertising does not pay the advertiser, for the reason that it offends that sense of propriety innate in us. It does not pay because it bespeaks overassurance, conceit or self-laudation. We all understand the difference between a business and a profession well enough to know that when a business man advertises, he praises his goods as being of superior quality; but when a member of a profession advertises, he asserts his own superior qualifications over those of his competitors. The business man lauds his goods, while the advertising dentist lauds himself.

He speaks of his superior skill in the practice of his profession, and this to the cultivated class of any community cannot help but be an offense. The man who says in public or in private that he is more competent to perform good dental operations than his competitors is only hurting himself, for the public soon judges the man at his proper merit and the assertion of superiority, even if strictly true in every sense, reacts upon the man who ventures the assertion. This is even more true of the man who puts a cheap price on his work, for as we value ourselves so shall others value us.

Though this kind of advertising is looked down upon by our better dentists, there still remains in a good many minds the impression that it pays financially. I am firmly convinced that this is a great mistake and by not having been combated long ago has been the means of many a promising man starting into practice upon the wrong road. He sacrifices his personal tastes in the hope of more speedily gaining a competence. He has heard a good deal of high stilted denunciation of advertising by older dentists, but he thinks he can easily see why they do not want the younger man to advertise. The older ones already have the practice and are afraid of being supplanted by the young men if the latter use this method. Their treatment of the young men encourages this idea, for in college and society nothing on the subject can be heard but denunciation and vilification. The subject is never calmly discussed and reason brought to play upon it. Here is where the colleges fail in their duty. They substantially tell the student that advertising pays well, but it is a crime and the person not respectable who resorts to it. This arouses the antagonism of the student, for common sense tells him it is not a crime. The student should not be told that advertising is a crime; he should be told that it shows bad taste, and he emphatically should be made to see that it does not pay to resort to it.

Dentists should be made to see that when they advertise, they are making a bid for the practice of the undesirable, the uncultivated aud the ignorant, and are building around themselves an obstacle which prevents them from gaining the practice of the better class. By the term "better class" I do not mean the wealthy class exclusively. A practice among the wealthy people is generally composed of patients of culture and refinement, but there are many wealthy people who are very undesirable patients.

On the other hand, a practice among the poorer classes is likely to be composed largely of overexacting, suspicious or ignorant patients, though many poerer people make most desirable patients. We, therefore, should not estimate the class of practice by the amount of money the patients possess, but rather should we estimate it by the amount of education, cultivation and appreciation of those patients.

A dentist, then, in striving for a first class practice should try to secure the patronage of this, as I call it, better element, and the well-to-do-people will be his when he has secured that element. The advertising that does not pay is the kind that disgusts this cultivated element.

The education of the public, dental as well as general, is making vast strides in these days, and a dentist cannot afford to content himself with a practice of the undesirable class while his competitors walk away with the constantly enlarging class of the cultivated and desirable.

The class of patients who are drawn by advertising are the class who from lack of intelligence and cultivation are unable to properly appreciate the best efforts of the dentist, and what man without daily encouragement can do his best ?

When a dentist's best efforts are unappreciated; when he seems to be making more money and with less effort from his poor work than he does from his best work—that dentist will not long continue to make his best efforts.

Suppose we imagine, for example, two young men who have graduated and started into practice. They are of equal ability and have both good chances for success in life. Let the first one be in a hurry to secure his practice. He is impatient and cards the papers. At first he makes no unjust claims to superiority over his competitors, but he soon sees that a plain card is doing him no good. He must have something startling to announce in order to attract the attention, or some extravagant cut to draw the eye. On one side of his plain card in the paper is a skeleton hideous to see, which represents to people what they will become if they do not take those world-famous little liver pills that have already saved untold thousands from the yawning grave. On the other side of his card is something equally glaring and inconsistent. In such surroundings his card does not show up, so he is compelled to change it.

He now inserts the cut of a set of teeth and underneath says, "Best for $5," or he claims to be the only dentist in the city capable of performing operations in crown-and bridge-work that are up to the standard required by modern methods. Now his card attracts notice and he draws the patronage of the same class of people who when they see the skeleton and read of the wonderful pills immediaiely buy a box of them.

The second young man does not rely on the papers to secure his patronage. The people who are attracted to him by his quiet, gentlemanly demeanor are people who are able to appreciate and value the best efforts he can make.

At the end of a period of ten years what would we see changed about these young men, provided they had conducted their practices through that term of years as they had begun? Have they advanced in the world and retained the good opinion of their community? The second young man has advanced without doubt, and when the residents are asked by strangers to be directed to the dentist who does the best work, they are sent to him. He is prosperous and in enjoyment of a lucrative practice among the best people.

The first young man may have advanced some financially. He still has his practice, but it is not of the better people and much of his income he is compelled to expend in advertising in order to retain the practice he has. Whenever a stranger asks for the dentist who will do his work the cheapest, he is directed to this young man. In professional attainments, in manual skill and ability he has retrograded, whereas his competitor each year has become more competent.

There may be gentlemen here who will not entirely agree with me in this statement, but I am confident there never was a dentist who made even a financial success by advertising. When you see a man who has made his way in the world by the practice of dentistry in a quack way, you may think that his success is because of his quackery, but I am sure it is in spite of it. There can not be a greater millstone around a dentist's neck than this quackery; and the man who has succeeded with it has in him elements of strength that would have brought to him much greater success had he not burdened himself with this Juggernaut. A dentist cannot be at his best for any length of time if he works on patients who have not the culture to appreciate his best work or who have not the money to properly pay for it. The encouragement and stimulation derived from our patients is something our inner self so keenly feels that its effect on us, whether we recognize it or not, is incalculable. To have a patient for whom you have done some difficult piece of operating compliment you upon your skill and thank you for the effort you have made does the ordinary man more good than the fee he receives for the case, provided he himself feels that the praise is deserved.

There is another class of advertising where the offense is not so evident, and this method is resorted to by many dentists who are supposed to be above reproach on this score. This class is none the less to be condemned because its source is concealed. It makes little difference whether you flatter yourself or whether you pay some one else to flatter you. The fault is yours in either case. This difference, however, does exist, that whereas the first kind of advertising spoken of will not pay, this latter kind will pay if it is done in a shrewd manner, for the less the advertisement seems to emanate from the dentist himself the more is it likely to draw to him patients who are desirable.

We all know cases where this kind of advertising is resorted to. One method employed is to have the newspaper reporter call on you and give you a little write-up, telling of the extensive improvement you are making in your office outfit. Another method is to give a lecture or a series of lectures in the public schools on dental subjects. The dental education of the people is an object we all wish to see accomplished, but we cannot help but see that the lecturer at the same time is receiving a splendid advertisement. The same is true of the writer of a treatise on care of the teeth, who has his article published in the papers. Better advertisements than these cannot be had for love or money. Among other ways I may mention the man who joins several lodges and possibly causes himself to be elected an officer in one or more of them for the business influence it may give him. Also the man who was once a demonstrator in a college and whose professional card forever after tells of the fact. And worst of all, the man who will join church for reasons he cannot publicly announce. He becomes regular in his attendance at prayer meeting; at the class-meeting and missionary societies he is always present and receives his periodical advertising. This I believe is seldom done, but there is no doubt that it has been practiced, disgusting as it is to a man of any character.

There is one point about this class of advertisers to which I wish to call attention. A good many, if not the majority of them, are men who have subscribed to the code of ethics, and some of them are prominent in society work. The evident reason is that it can be done without breaking the letter of the code. The spirit of it is violated in every instance as much as is done by the much-abused quack. They have the consistency of the man who says it is all right to steal, but a great crime to be caught at it. They wear the mark of the hypocrite. They love to pray standing in public places, yet at other times will do things which ethically considered are shady, to say the least. In this particular you can see that the code is a failure. It catches the small fry, the men who adopt the least remunerative advertising, while the bigger fish, the ones who makes the most profit from their advertising, are not interfered with.

I have heard two methods advocated for the cure of this. One is to make the code more stringent. It is proposed to make the code so strict that all kinds and degrees of fish will be caught. I think this would be a very hard if not an altogether impossible thing to do. I do not think a dentist can be made a professional man by written code of ethics, no matter how stringent may be its rules. Law will never make a respectable man of a disreputable one, and a code will never make a professional man of a quack. It never has and it never will.

The second method proposed is to abolish the code entirely, and in this measure I see the bettering of our profession. The foremost advocate of this procedure is Dr. Ottolengui, the present editor of the *Items of Interest*, and this fact itself speaks well for it.

There are a great many men who know that they have a perfect legal right to advertise, and think that associations, by adopting codes of ethics which reflect upon the conduct of the advertiser, are exceeding their authority. They look upon the association as a band of men who think themselves morally superior to every one else, as a mutual admiration society, which is only an exhibition of the old spirit of the Pharisee.

It may be answered that any society has a right to adopt laws to govern its own members, and society members may have a code if they wish without causing offense to non-members. We must remember that the code, however, is not a law for the society, but it is a law drawn up by the society which states that the duty of all dentists, whether society members or not, is to obey that law. Those not obeying are stigmatized as quacks. Here in Indiana we have possibly 600 dentists, less than one-sixth of whom have subscribed to a code of ethics, yet this one-sixth sets itself up in judgment over the other five-sixths. The spirit of opposition and resentment felt by every liberal-minded man; whether he is an advertiser or not, has been and is to day the means of so dividing the members of our profession that a universal effort towards a common good is impossible. The code has in it the elements of a force bill. It does not correspond well with the enlightened spirit of the day. Dentistry is classed with the liberal professions. It is surely time for some liberality to be introduced into our relations to each other. Let us be liberal in spirit as well as in name. Let us recognize the fact that force as a moral persuader with a man of education is not a success. A man of intelligence must be led by means of his reasoning power. The majority of an enlightened profession cannot be dictated to by a minority. The dental profession to-day is composed of men of intelligence and mental ability, and not of a pack of school-boys who must be controlled by a switch.

Threats and denunciations as measures in controlling men's opinions are not successful. They belong to a former age. In no other place is this more plainly shown than in religious teaching. The old-time preacher used to argue, "Lead a good life, or you will eternally turn in hell." Some of our good brother revivalists are still too much on this order, but it is fast passing away. The argument to-day most used by the successful minister is, "Lead a good life, and you will find that it pays in this world." It pays every day, financially, morally and physically. Men can see the truth of this, and are attracted by it, who by the old argument would only be disgusted. The religion has not changed. It is the same old Christianity, but the teaching of it has changed and must continue to change as long as man grows and develops intellectually and morally.

The code of ethics has seen its day, if it ever had one, and it should be cast off as we would cast off a worn-out garment. We will always have advertisers in the profession, among societies' members as well as among non-members, but there is no power in a code of ethics to correct the evil.

Eminent legal opinion is almost a unit in favor of the repeal of a law that cannot be enforced. The code is a dead letter. It not only cannot be enforced, but it works a positive injury to us. It divides us into two opposing factions and creates bad blood. Let us adopt a more efficacious method. Instead of bigotry, let us have liberality; instead of empty denunciation, let us have convincing argument.

There is one test of the code of ethics I would have you apply. Ask the dentists who have led and are leading professional lives if they do so because of the code of ethics, and in a hundred of them every one will say, "No; I observe the spirit of the code because it is right. I believe it is the manly and the proper thing to do. I believe it pays me every day to observe it, and if it were abolished, my conduct would not change one iota."

Here is evidence that is indisputable. Evidence that inevitably convicts the code and convicts it as worthless on the evidence of its own supporters. We know it does not influence the non-members of societies and we see that it does not influence society members themselves. Then pray tell me whom it does influence?

The growth and success of the Eastern Indiana Dental Association can, to a large extent, be ascribed to the fact that it has solved this question. It holds out the hand of good fellowship to all, whether high or low, and shuts the door in the face of no one on account of mistakes he may have made or may be making to-day. It recognizes the fact that the best way to reform a man is to place in company which will have the right influence on him. It allows every man to regulate his conduct according to his own conscience, and does not attempt to pass judgment upon those whe do not come up to the highest ethical standard.—*Indiana Dental Journal.*

